# Atomistically Informed Extended Gibbs Energy Description for Phase-Field Simulation of Tempering of Martensitic Steel

**DOI:** 10.3390/ma9080669

**Published:** 2016-08-09

**Authors:** Oleg Shchyglo, Thomas Hammerschmidt, Miroslav Čak, Ralf Drautz, Ingo Steinbach

**Affiliations:** Interdisciplinary Centre for Advanced Materials Simulation (ICAMS), Ruhr-Universität Bochum, Universitätsstr. 150, Bochum 44801, Germany; thomas.hammerschmidt@rub.de (T.H.); miroslav.cak@rub.de (M.Č.); ralf.drautz@rub.de (R.D.); ingo.steinbach@rub.de (I.S.)

**Keywords:** martensite, steel, elasticity, CALPHAD

## Abstract

In this study we propose a unified multi-scale chemo-mechanical description of the BCT (Body-Centered Tetragonal) to BCC (Body-Centered Cubic) order-disorder transition in martensitic steel by adding the mechanical degrees of freedom to the standard CALPHAD (CALculation of PHAse Diagrams) type Gibbs energy description. The model takes into account external strain, the effect of carbon composition on the lattice parameter and elastic moduli. The carbon composition effect on the lattice parameters and elastic constants is described by a sublattice model with properties obtained from DFT (Density Functional Theory) calculations; the temperature dependence of the elasticity parameters is estimated from available experimental data. This formalism is crucial for studying the kinetics of martensite tempering in realistic microstructures. The obtained extended Gibbs energy description opens the way to phase-field simulations of tempering of martensitic steel comprising microstructure evolution, carbon diffusion and lattice symmetry change due to the ordering/disordering of carbon atoms under multiaxial load.

## 1. Introduction

The strong dependence of mechanical properties of martensitic steel on the heat treatment is well established and widely used to tune the materials properties during processing. The formation of martensite is a diffusionless phase transformation, where the chemical degrees of freedom are static and almost inactive during the transformation. As the result of such a transformation the quenched martensitic steel has limited toughness. The situation changes if martensite is subjected to heat treatment at temperatures below the austenitization temperature, a process which is typically called tempering. In such a case, carbon, as the most mobile alloy component in carbon steel, starts to rearrange on the interstitial sublattice to minimize its energy. This has significant effect on the material’s final properties. There are several phenomena which contribute to the tempering effect, e.g., carbide formation, recovery, carbon redistribution and corresponding lattice parameter change, leading to an increased toughness of the heat treated martensitic steel.

**Figure 1:**
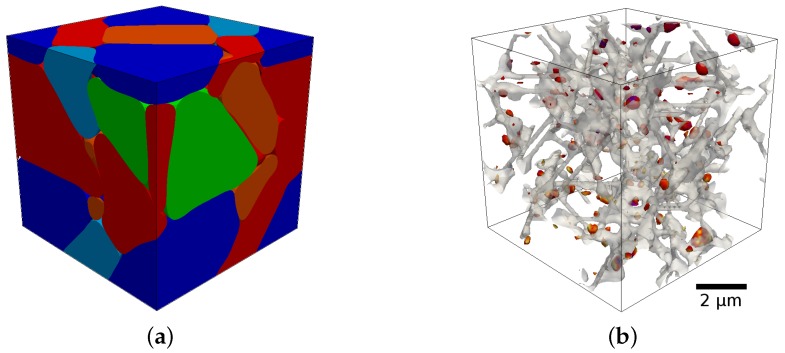
Typical microstructures of as-quenched (**a**) and tempered (**b**) martensite in carbon steel obtained using the phase-field method with chemo-mechanical coupling. The as quenched martensite microstructure considers all 24 symmetry related martensite variants following the Kurdjumov-Sachs orientation relationship. The tempered martensite microstructure shows only the interface regions filled with the carbides particles. For further details see [1].

Since all these phenomena appear in the solid material, significant mechanical relaxations are expected due to the change of the lattice parameter stemming from the phase transformation and carbon redistribution. This makes the coupling of chemical and mechanical degrees of freedom an important contribution to the thermodynamic phase stability in the solid state. We integrate this by an atomistically informed multi-scale approach where elastic deformations of the crystal lattice are derived from a phase-field simulation of the martensitic transformation. A CALPHAD type sublattice model is used to describe the carbon activity in the distorted lattice and its effect on the transformation kinetics. Figure 1 shows an example of such a phase-field simulation of the tempering of martensite using the coupled chemo-mechanical approach [1]. For details on how to integrate a sublattice model into the phase-field framework see [2]. For the phase-field framework in general see [3,4].

In order to incorporate the mechanical energy contribution to the thermodynamic stability analysis of the martensite we start from the standard CALPHAD type description of alloy thermodynamics. The CALPHAD method is a well established approach providing Gibbs energies of the thermodynamic phase in the form of databases, where the composition and temperature dependence of Gibbs energies is modeled using the compound energy formalism [5]. The contribution of the mechanical degrees of freedom is modeled through the hydrostatic pressure dependence in the CALPHAD type description. While such an approach is well justified for the modeling of bulk phases at normal conditions, the modeling of solid phases within the microstructure of real materials requires consideration of the multiaxial load, the full set of elasticity parameters of solid phases including their lattice symmetry due to the non-negligible stresses from the different orientations and lattice symmetries in the microstructure. This information will be provided locally within each reference volume of the phase-field simulation domain.

In order to account for the mechanical degrees of freedom and their dependence on the alloy composition, we introduce the elastic energy contribution to the Gibbs energy using the composition dependent crystal lattice parameter and elasticity moduli following the approach proposed in [6].

## 2. Crystallography of Ordered BCT and Disordered BCC Phases in Fe-C System

The initial appearance of the ordered BCT martensite crystal lattice is due to the fast transformation from the austenite to the martensite which traps the carbon atoms on the particular subset of interstitial sites. Thus, the initial arrangement of carbon atoms and the corresponding lattice symmetry of martensite is determined by the austenite-martensite transformation path. Zener [7] proposed that such a structure is not only the result of the trapping of carbon atoms but that it also corresponds to a stable crystal structure. Recently, an alternative ordered BCT structure, the $\alpha^{\prime \prime }$-phase with Fe_16_C_2_ stoichiometry, was proposed [8]. The motivation for the newly proposed $\alpha^{\prime \prime }$ BCT ordered structure is the absence of evidence of Zener-ordering in experiments which otherwise would be identified as local clusters with carbon concentration of around 50 at.%. In contrast, experiments show much lower concentrations for carbon-rich sites of the order of 11 at.% which is close to the Fe_16_C_2_ stoichiometry [9]. The thermodynamic stability analysis [8] shows two competing tendencies at room temperature: order-disorder transition from the ordered BCT phase to the disordered BCC phase and the phase separation within the ordered structure itself. The same tendency holds for both the classical Zener-ordered $\alpha^{\prime }$-phase and the newly proposed $\alpha^{\prime \prime }$-phase. While both, the $\alpha^{\prime }$-phase and $\alpha^{\prime \prime }$-phase, show the same tendency, the order-disorder transition temperatures are significantly different with the $\alpha^{\prime \prime }$-phase being stable up to higher temperatures than the $\alpha^{\prime }$-phase. In technological systems where the tempering of martensite is a key process to tailor the materials properties, such a difference is of key importance because it will directly affect the tempering kinetics.

**Figure 2:**
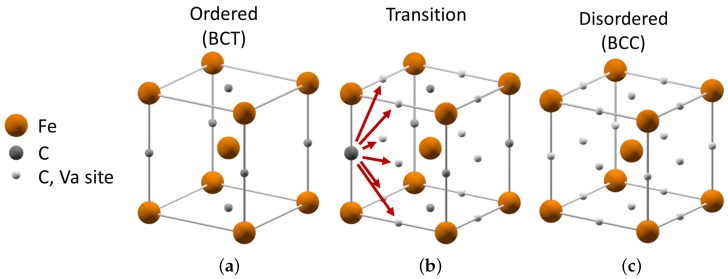
Crystal structure of ordered BCT $\alpha^{\prime \prime }$-phase (**a**); disordered BCC *α*-phase (**c**); and the transition path between them (**b**) in the Fe-C system with vacancies (Va). The transition from ordered to disordered carbon atoms can be accomplished by moving the carbon atoms to nearest neighbor sites.

The order-disorder transformation path is illustrated in Figure 2. It indicates the set of crystal structures involved in the transformation from ordered BCT $\alpha^{\prime }$- or $\alpha^{\prime \prime }$-phase to disordered BCC *α*-phase in plain carbon steel. There are multiple ways which lead from ordered to disordered carbon distribution as shown in the middle structure in Figure 2. The arrows indicate the ways in which the carbon atom can change its position and the corresponding symmetry of its lattice site. If an uncorrelated motion on the nearest neighbor distances of the large number of carbon atoms takes place, the resulting crystal structure becomes disordered. In case of a correlated motion of carbon atoms, the entire crystal can change its symmetry from one ordered arrangement to another. Both tendencies can be mediated by the local lattice distortions in real martensitic microstructures subjected to heat treatment.

## 3. Atomistic Modeling of Elastic Properties

The CALPHAD type approach to a chemo-mechanical description requires knowledge of the lattice parameters and elastic moduli of the considered phases. Due to the lack of experimental data, we determined these data for the $\alpha^{\prime \prime }$-phase by density-functional theory (DFT) calculations. The calculations were performed with the VASP package [10,11] in combination with a high-throughput environment [12]. We used the projector augmented wave (PAW) pseudopotentials [13] and the Perdew-Burke-Ernzerhof (PBE) functional [14] within the generalized gradient approximation to the exchange-correlation functional. All calculations were spin-polarized with a plane-wave cutoff of 500 eV and a Monkhorst-Pack k-point mesh [15] with a density of 0.02 ${Å}^{3}$. The structures were fully relaxed (i.e., atomic positions and unit cells) until the maximum force was below 0.01 eV/Å. The elastic constants were determined by applying a strain tensor to the simulation cells as described in previous works on interstitial H in BCC-Fe [16] and substitutional B in BCC-Fe [17].

**Figure 3:**
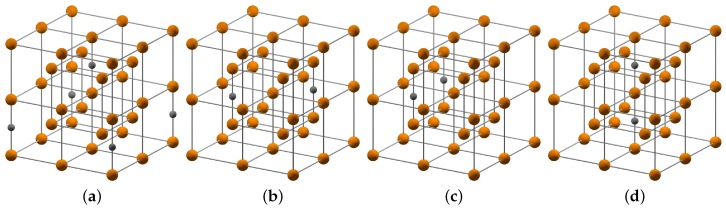
Simulation cells of $\alpha^{\prime \prime }$-${\mathrm{Fe}}_{16}C_{2}$ with different arrangements of carbon atoms (black) in the BCC lattice of Fe atoms (brown) used in the density-functional theory (DFT) calculations. The ordering of configuration I (**a**) to configuration IV (**d**) reflects the energetic ordering of most stable to least stable (cf. Table 1).

The $\alpha^{\prime \prime }$-phase corresponds to a chemical composition of Fe_16_C_2_ with carbon in interstitial positions of a BCC Fe lattice. The distribution of carbon atoms in the sublattice of interstitial sites can vary with temperature and processing history. In order to mimic the limiting cases, we considered a representative set of simulation cells with different carbon distribution as shown in Figure 3. The considered carbon distributions cover the smallest and largest possible distance of two carbon atoms as well as configurations with and without an iron atom in between two carbon atoms.

The results of our DFT calculations for the carbon distributions I–IV are compiled in Table 1, together with the results for carbon-free *α*-Fe. Our results show that the configuration with a tetragonal unit cell and the largest carbon-carbon separation (configuration I) is energetically most favorable. The tetragonal unit cell with an iron atom between two carbon atoms in [110] direction (configuration II) corresponds to a chain of carbon atoms perpendicular to the tetragonal distortion in [001]. This configuration is predicted to be only 8 meV/atom higher in energy. The orthorhombic unit cell without an iron atom between two carbon atoms (configuration III) corresponds to a carbon chain along the [100] direction and is 17 meV/atom higher in energy than configuration I. The tetragonal unit cell with an Fe atom between two carbon atoms in [001] direction (configuration IV) is the least stable configuration with 77 meV/atom above configuration I. The tetragonal distortion of the relaxed unit cells in [001] is similar for the configurations I–III. The configuration with a carbon chain along [001], however, leads to a significantly increased tetragonal distortion and to mechanical instability ($C_{66}<0$). The computed formation energies indicate that the carbon configurations I–III of $\alpha^{\prime \prime }$-Fe_16_C_2_ can be expected at room temperature. For the purpose of this work we consider only configuration I in the chemo-mechanical description as the energetically competing configurations II and III have comparable lattice constants and elastic moduli.

## 4. Elastic Energy Contribution to the Gibbs Energy

In order to include the elastic energy in the standard Gibbs energy description, we start from the standard Gibbs energy models of *α*-Fe and $\alpha^{\prime \prime }$-Fe phases and add the elastic energy terms

(1) GBCC=GBCCch+GBCCelGBCT=GBCTch+GBCTel

to the standard Gibbs energy models GBCCch [18] and GBCTch [8]. The elastic energy terms, GBCCel and GBCTel, are given by

(2) GBCCel=νBCC2ϵCBCCϵGBCTel=νBCT2ϵCBCTϵ

where $\nu^{BCc}$ and $\nu^{BCT}$ are the equilibrium molar volumes of the phases and $C^{BCC}$ and $C^{BCT}$ are the composition and temperature dependent elasticity tensors of the BCC and BCT phases, *ϵ* is the elastic strain tensor representing the lattice distortion resulting from the mechanical relaxation as given from the external phase-field simulation (one-way coupling). The minimized sublattice model defines the driving forces of the transformation and diffusion in the phase-field simulation (two-way coupling). The description of the elastic moduli of the BCC *α*-Fe and BCT $\alpha^{\prime \prime }$-Fe phases follows [6]

(3) $$\begin{array}{ccc} C^{BCC} & = & y_{Fe}^{I}y_{Va}^{II}C^{Fe:Va}+y_{Fe}^{I}y_{C}^{II}C^{Fe:C}\\ C^{BCT} & = & y_{Fe}^{I}y_{Va}^{II}y_{Va}^{III}C^{Fe:Va:Va}+y_{Fe}^{I}y_{C}^{II}y_{Va}^{III}C^{Fe:C:Va}+y_{Fe}^{I}y_{C}^{II}y_{C}^{III}C^{Fe:C:C} \end{array}$$

where the $y_{A}^{N}$ are the site fractions of the elements (A=Fe,C and vacancy Va) on the sublattices (N=I,II and III). The temperature dependence of the elasticity parameters of end members, $C^{Fe:Va}$, $C^{Fe:C:C}$, $C^{Fe:Va:Va}$, $C^{Fe:C:Va}$ and $C^{Fe:C:C}$ is assumed to be linear. A more detailed discussion of this choice is presented in Section 5. The lattice parameters of the BCC and BCT phases are given by

(4) $$\begin{array}{ccc} a^{BCC} & = & y_{Fe}^{I}y_{Va}^{II}a^{Fe:Va}+y_{Fe}^{I}y_{C}^{II}a^{Fe:C}\\ a^{BCT} & = & y_{Fe}^{I}y_{Va}^{II}y_{Va}^{III}a^{Fe:Va:Va}+y_{Fe}^{I}y_{C}^{II}y_{Va}^{III}a^{Fe:C:Va}+y_{Fe}^{I}y_{C}^{II}y_{C}^{II}a^{Fe:C:C}\\ c^{BCT} & = & y_{Fe}^{I}y_{Va}^{II}y_{Va}^{III}c^{Fe:Va:Va}+y_{Fe}^{I}y_{C}^{II}y_{Va}^{III}c^{Fe:C:Va}+y_{Fe}^{I}y_{C}^{II}y_{C}^{III}c^{Fe:C:C} \end{array}$$

where $a^{Fe:Va}$, $a^{Fe:Va:Va}$ and $c^{Fe:Va:Va}$ are identical lattice parameters of pure *α*-Fe, $a^{Fe:C}$, $a^{Fe:C:C}$ and $c^{Fe:C:C}$ are identical and equal to the lattice parameter of the BCC Fe-C structure in which all interstitial sites are filled by carbon atoms. The lattice parameters $a^{Fe:C:Va}$ and $c^{Fe:C:Va}$ correspond to the lowest energy BCT crystal structure with Fe_16_C_2_ stoichiometry (cf. Section 3). For the lattice parameters in Equation (4), we consider a linear temperature expansion as discussed in Section 5.

## 5. Results

In order to include the temperature dependence of the elastic moduli we compare our data given in Section 4 and the pure iron elastic constants from [19]. For simplicity we assume a linear temperature dependence of the elasticity moduli

(5) $$\begin{array}{ccc} C^{A}\left(T\right) & = & C_{T=0K}^{A}\circ \left(I+\Gamma_{T}T\right) \end{array}$$

where (∘) indicates the Hadamard product, I is the tensor with all components equal to unity, $\Gamma_{T}$ is the tensor of linear temperature dependence coefficients and $C_{T=0K}^{A}$ are the DFT calculated elastic tensors of the end member structures of the sublattice model (see Table 1). For consistency we scale down our elasticity data by 11% to match the experimentally observed values and applied the same scaling factor to all end members assuming that the relative elastic behavior of the phases follows the same trend. In this paper we assume the same temperature dependence of elasticity moduli of both *α*-Fe and $\alpha^{\prime \prime }$-Fe phases. The components of the linear temperature dependence tensor, $\Delta C_{T}$, evaluated using the data from [19], are given in Table 2.

Due to the lack of experimental data on the thermal expansion coefficients of the $\alpha^{\prime \prime }$-Fe phase, we assume them to be the same as the thermal expansion coefficients of the *α*-Fe phase. Since only ratios of the lattice parameters enter the elastic energy calculations, the identical linear temperature dependence terms cancel out. Thus, it is sufficient to consider the relative differences of the lattice parameters of both phases at T=0K which are given in Equation (4) as it is done in further analysis in this study.

Employing the elastic data in the Gibbs energy model (Equation (1)) we obtain the coupled atomistically-informed chemo-mechanical model of the *α*-Fe and $\alpha^{\prime \prime }$-Fe phases suitable for tempering kinetics simulations using the phase-field method.

**Figure 4:**
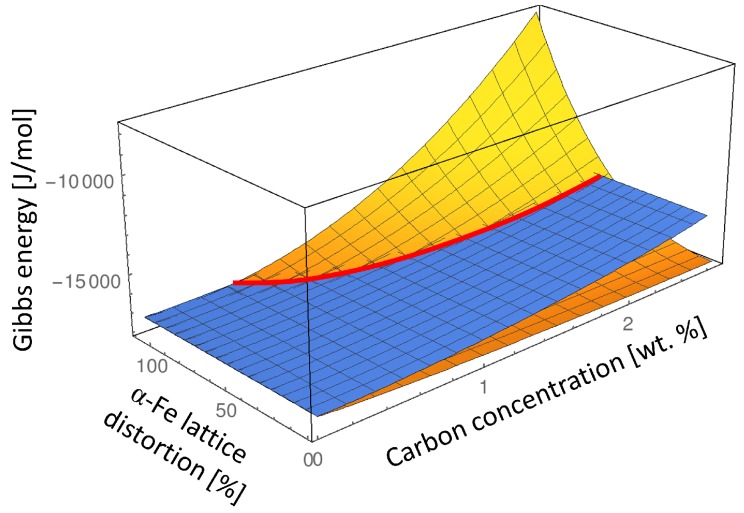
Intersection between the Gibbs energies of $\alpha^{\prime \prime }$-Fe (blue) and *α*-Fe (yellow) at T=550 K while gradually distorting the *α*-Fe lattice from its equilibrium state (0% *α*-Fe lattice distortion) to the maximum distortion given by Equation (6) (100% *α*-Fe lattice distortion). The lattice parameter of the $\alpha^{\prime \prime }$-Fe phase is kept constant at its equilibrium value for every composition (see Equation (4)).

In order to analyze the influence of carbon composition of the $\alpha^{\prime \prime }$-Fe phase on the $\alpha^{\prime \prime }$-Fe – *α*-Fe order-disorder transition, we determine the Gibbs energies of the ordered $\alpha^{\prime \prime }$-Fe and disordered *α*-Fe phase in composition and lattice parameter space at T=550 K which is typically the lowest martensite tempering temperature. In Figure 4 we keep the lattice parameter of the parent martensite $\alpha^{\prime \prime }$-Fe phase at its equilibrium value (see Equation (4)) and subject the *α*-Fe lattice to the elastic strain ranging from zero to the maximum lattice misfit strain between the $\alpha^{\prime \prime }$-Fe and *α*-Fe phases:

(6) $$\begin{array}{c} \epsilon^{max}=\left(a^{BCT}-a^{BCC}\right)/a^{BCC} \end{array}$$

Since the value of $\epsilon^{max}$ is a function of composition due to composition dependence of the lattice parameters $a^{BCC}$ and $a^{BCT}$ it is presented in % for each composition in Figure 4.

Analyzing such an energy landscape allows us to illustrate the transformation path from the ordered $\alpha^{\prime \prime }$-Fe phase to the disordered *α*-Fe phase which takes into account not only the chemical energy difference but also considers the elastic strain effect. According to Eshelby’s elliptic inclusion analysis [20] if a small inclusion with different lattice parameter is introduced into the infinite matrix material, the most of the misfit strain and thus the elastic energy will be concentrated inside the inclusion. This means that if a nucleus of the disordered *α*-Fe phase would nucleate inside of the ordered $\alpha^{\prime \prime }$-Fe matrix phase it would initially have the lattice parameters close to the lattice parameter of the $\alpha^{\prime \prime }$-Fe matrix phase. Thus, the approximate transition path from the ordered $\alpha^{\prime \prime }$-Fe to the disordered *α*-Fe phase can be read from Figure 4 by taking the Gibbs energy value of the ordered $\alpha^{\prime \prime }$-Fe matrix phase in its equilibrium lattice and the Gibbs energy of the emerging *α*-Fe phase subjected to the maximum elastic strain given by Equation (6) (indicated as 100% *α*-Fe lattice distortion in Figure 4).

**Figure 5:**
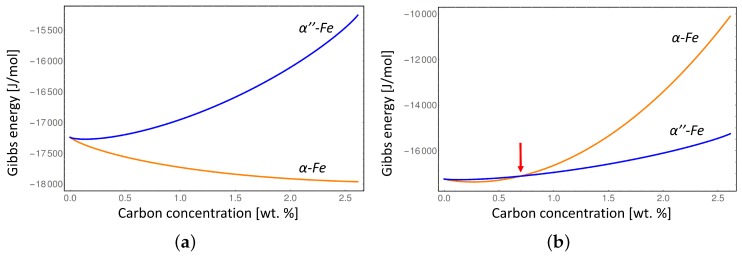
Gibbs energies of *α*-Fe and $\alpha^{\prime \prime }$-Fe at T=550 K considering their equilibrium lattices (**a**) and considering maximum lattice distortion of the *α*-Fe phase (**b**).

Figure 5 shows the comparison between the Gibbs energies of *α*-Fe and $\alpha^{\prime \prime }$-Fe phases at two extremes: in their equilibrium lattices which corresponds to zero elastic energy contribution to the Gibbs energy of both phases (0% *α*-Fe lattice distortion in Figure 4), and considering the maximum lattice distortion of the *α*-Fe phase while keeping the lattice parameter of the $\alpha^{\prime \prime }$-Fe at its equilibrium value which corresponds to the nucleation condition of the *α*-Fe phase and results in the maximum elastic energy contribution to the Gibbs energy of the *α*-Fe phase (100% *α*-Fe lattice distortion in Figure 4).

The conventional Gibbs energies considering equilibrium lattice parameters of the *α*-Fe and $\alpha^{\prime \prime }$-Fe phases (Figure 5a) show that the disordered *α*-Fe phase is stable at all compositions of carbon at T=550 K, while considering the *α*-Fe lattice distortion (Figure 5b) indicates that *α*-Fe is only stable below approximately 0.7 wt % due to the misfit strain effect. In addition, the intersection between the Gibbs energies with elastic energy contribution (red line in Figure 4, and red arrow in Figure 5b) indicates the degree of local deformation of the $\alpha^{\prime \prime }$-Fe matrix necessary to stabilize the disordered *α*-Fe phase. It is clear that at certain carbon composition the elastic deformation limit of $\alpha^{\prime \prime }$-Fe will be exceeded during the transition to *α*-Fe leading to the plastic deformation. Since we can not address the plastic deformation in a generic way in this study we limited our consideration to the elastic deformation only. Thus our results represent the maximum value of the elastic energy contribution which would typically be lower at higher carbon concentrations due to plastic relaxation.

**Figure 6:**
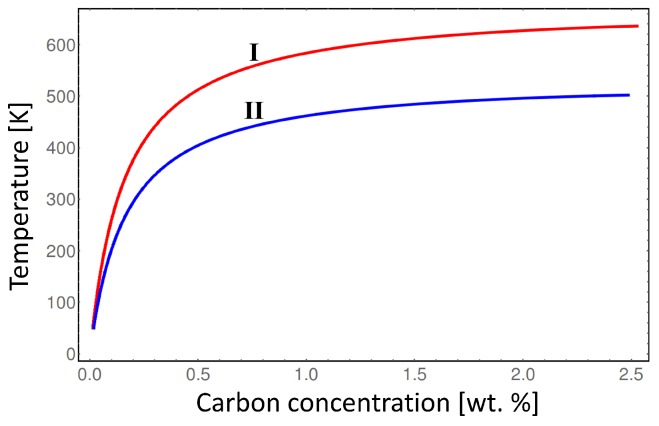
Comparison of the order-disorder transition temperature between *α*-Fe and $\alpha^{\prime \prime }$-Fe phases considering the deformed state of the *α*-Fe phase (I) and considering both phases in their equilibrium lattices (II).

In order to overcome the elastic energy barrier, the system should be heated to higher temperatures to provide enough chemical driving force to compensate for the elastic strain effect. Figure 6 shows, that considering elastic energy contribution, the order-disorder transition temperature between *α*-Fe and $\alpha^{\prime \prime }$-Fe phases gradually rises by almost 150 K with the increasing carbon content compared to the case without the chemo-mechanical coupling. Note, that the result presented in Figure 6 represents the maximum order-disorder transiton temperature deviation due to elastic energy contribution because it assumes maximum deformation of the *α*-Fe phase compared to $\alpha^{\prime \prime }$-Fe phase. In the real microstructure the non-uniform distortion of the parent $\alpha^{\prime \prime }$-Fe phase will result in lower order-disorder transition temperature than indicated by the line I in Figure 6. Thus, in order to investigate in detail the order-disorder transition kinetics during martensite tempering a realistic microstructure simulation should be perfomed considering carbon diffusion and mechanical relaxation of the crystal lattice. This can be done by means of phase-field method as presented in [1].

## 6. Conclusions

We introduce a unified multi-scale chemo-mechanical model that includes the mechanical degrees of freedom to the standard CALPHAD type Gibbs energy description. The proposed model takes into account the effect of carbon composition on the lattice parameter and elasticity moduli of *α*-Fe and $\alpha^{\prime \prime }$-Fe phases by a sublattice model that is parameterized on the basis of DFT calculations. The variation of elastic parameters with temperature are estimated from available experimental data.

The DFT calculations for the $\alpha^{\prime \prime }$-Fe_16_C_2_ phase indicate three competing carbon distributions with comparable lattice constants and elastic moduli. The energetically most favorable arrangement of carbon atoms is observed for a tetragonal unit cell with the largest considered separation of two carbons atoms. The two competing configurations correspond to chains of carbon atoms along the [110] and [100] direction, i.e., to directions perpendicular to the tetragonal distortion. A fourth configuration with a chain of carbon atoms parallel to the direction of the tetragonal distortion is considerably higher in energy and mechanically unstable. Preliminary analysis of the thermodynamic stability of *α*-Fe and $\alpha^{\prime \prime }$-Fe phases using the proposed model shows the significant effect of elasticity on the order-disorder transition temperature. The increase of the transition temperature due to elastic energy effect is in accordance with experimental observations where tempering starts at higher temperatures than it is predicted by the phase stability analysis if elastic energy effect is not considered. Moreover, in order to reveal the stability of phases comprising the real microstructures, two-way chemo-mechanical coupling is required. Such a coupling provides the mutual influence between the chemical and mechanical degrees of freedom and allows us to take into account the effect of elasticity on the diffusion kinetics as well as the effect of composition on the elastic properties, which directly affect the phase transformation kinetics. The two-way coupling is realized using the phase-field framework which seamlessly considers microstructure evolution, diffusion and mechanical relaxation of the system in one simulation. This enables us to study the kinetics of martensite tempering while considering realistic microstructures in a wide range of processing conditions. For application of the proposed model in phase-field simulation of martensite tempering see [1].

## Figures and Tables

**Table 1:** Lattice constants *a*, *b*, *c* and elastic moduli $C_{ij}$ of $\alpha^{\prime \prime }$-${\mathrm{Fe}}_{16}C_{2}$ with carbon distributions I–IV (cf. Figure 3) as obtained from DFT calculations. The corresponding values for *α*-Fe and *α*-FeC_3_ are given for comparison. The carbon distributions I-IV are ordered by relative energetic stability ΔE with respect to the most stable configuration I.

	*α*-Fe	*α*-FeC_3_	$\alpha^{\prime \prime }$-Fe_16_C_2_-I	$\alpha^{\prime \prime }$-Fe_16_C_2_-II	$\alpha^{\prime \prime }$-Fe_16_C_2_-III	$\alpha^{\prime \prime }$-Fe-Fe_16_C_2_-IV
*a* (Å)	5.67	7.86	5.66	5.62	5.63	5.07
*b* (Å)					5.66	
*c* (Å)			6.25	6.33	6.27	7.50
$C_{11}$ (GPa)	278.2	251.7	269.3	276.7	265.3	215.7
$C_{22}$ (GPa)					278.1	
$C_{33}$ (GPa)			297.5	289.9	288.4	238.5
$C_{12}$ (GPa)	147.8	204.1	144.3	143.3	128.5	171.1
$C_{13}$ (GPa)			149.4	151.9	153.5	148.2
$C_{23}$ (GPa)					137.1	
$C_{44}$ (GPa)	95.1	30.6	94.5	95.1	93.5	14.8
$C_{55}$ (GPa)					80.1	
$C_{66}$ (GPa)			123.1	123.1	99.0	–131.3
ΔE (meV/atom)			0	8	17	77

**Table 2:** The components of the elastic moduli linear temperature dependence tensor $\Gamma_{T}$, [$K^{-1}$].

$\gamma_{11}$	$\gamma_{33}$	$\gamma_{12}$	$\gamma_{23}$	$\gamma_{44}$	$\gamma_{66}$
–0.000267	–0.000267	–0.000274	–0.000274	–0.000192	–0.000192
